# Use of a portable system with ultrasound and blood tests to improve prenatal controls in rural Guatemala

**DOI:** 10.1186/s12978-016-0237-6

**Published:** 2016-09-13

**Authors:** Patricia Hanna Crispín Milart, César Augusto Diaz Molina, Ignacio Prieto-Egido, Andrés Martínez-Fernández

**Affiliations:** 1 Department of Obstetrics and Gynecology, Alcorcón Foundation University Hospital, Madrid, Spain; 2 TulaSalud - non-governmental organization, Alta Verapaz, Guatemala; 3 EHAS Foundation, ESTI de Telecomunicación, Ciudad Univesitaria s/n, 28040 Madrid, Spain; 4 Higher Technical School of Telecommunications Engineering, Rey Juan Carlos University, Madrid, Spain

**Keywords:** Prenatal care, Health information systems, Portable ultrasound, Developing countries, Maternal mortality, Neonatal mortality

## Abstract

**Background:**

Maternal and neonatal mortality figures remain unacceptably high worldwide and new approaches are required to address this problem. This paper evaluates the impact on maternal and neonatal mortality of a pregnancy care package for rural areas of developing countries with portable ultrasound and blood/urine tests.

**Methods:**

An observational study was conducted, with intervention and control groups not randomly assigned. Setting: Rural areas of the districts of Senahu, Campur and Carcha, in Alta Verapaz Department (Guatemala). The control group is composed by 747 pregnant women attended by the community facilitator, which is the common practice in rural Guatemala. The intervention group is composed by 762 pregnant women attended under the innovative Healthy Pregnancy project. That project strengthens the local prenatal care program, providing local nurses training, portable ultrasound equipment and blood and urine tests. The information of each pregnancy is registered in a medical exchange tool, and is later reviewed by a gynecology specialist to ensure a correct diagnosis and improve nurses training.

**Results:**

No maternal deaths were reported within the intervention group, versus five cases in the control group. Regarding neonatal deaths, official data revealed a 64 % reduction for neonatal mortality. A 37 % prevalence of anemia was detected. Non-urgent referral was recommended to 70 pregnancies, being fetal malpresentation the main reported cause.

**Conclusion:**

Impact data on maternal mortality (reduction to zero) and neonatal mortality (NMR was reduced to 36 %) are encouraging, although we are aware of the limitations of the study related to possible biasing and the small sample size.

The major reduction of maternal and neonatal mortality provides promising prospects for these low-cost diagnostic procedures, which allow to provide high quality prenatal care in isolated rural communities of developing countries.

**Trial registration:**

This research was not registered because it is an observational study where the assignment of the medical intervention was not at the discretion of the investigators.

## Plain English summary

Maternal and neonatal mortality figures remain unacceptably high worldwide and the problem is especially severe in rural areas of developing countries. These areas usually lack of resources and personnel to follow international protocols on prenatal controls, which are based on ultrasonography and blood and urine tests. This paper evaluates the impact on maternal and neonatal mortality of a pregnancy care package designed for rural areas of developing countries. The pregnancy care kit includes a portable ultrasound system, dried blood and rapid urine tests, and is provided to nurses, together with the training required to diagnose the most common problems during pregnancy. The research defines an intervention group composed by 762 pregnant women that received a prenatal control by nurses using the prenatal kit. The control group is composed by 747 women that received the usual prenatal control in rural areas (by community facilitators without ultrasonography nor laboratory tests). The results show that no maternal deaths were reported within the intervention group, versus five cases in the control group. Regarding neonatal deaths, official data revealed a 64 % reduction for neonatal mortality when comparing the intervention group with the control group. This major reduction of maternal and neonatal mortality provides promising prospects for these low-cost diagnostic procedures, which allow to provide high quality prenatal care in isolated rural communities of developing countries.

## Background

Well into the XXI century, maternal and neonatal mortality figures remain unacceptably high worldwide. Only in 2013, 289,000 women died due to complications during pregnancy and childbirth, and about 2.8 million newborns died during their first month of life [1, 2].

According to the commitment of the United Nations through the Millennium Development Goals, maternal mortality should be reduced by 75 % by 2015; however, 2015 report [3] shows that in 2013 only 45 % of it had been achieved (reducing maternal mortality ratio -MMR- from 380 to 210). Regarding child mortality, whereas the commitment was a two third reduction, in 2012 it had only reached 70 % of that value (from 90 to 48 deaths per 1,000 live births). It should also be noted that almost half of these under five-year-old deaths occur during the neonatal period (before reaching 28 days) [3].

An important fact to understand this problem is that in developing countries, there were 40 million births without skilled health staff assistance; and more than 32 million of these births occurred in rural areas [3], where only one in every three women with an obstetric emergency received attention on time [4].

Several authors argue that the strategies to address this problem must combine wider coverage of the target population [5], timely emergency obstetric attention, and an increase in the overall quality of women care [6]. Although developing countries have increased the antenatal care coverage in the last years, the quality of these controls is still poor. Ultrasound scans and laboratory tests are essential prenatal care tools in high-income countries, but not yet widespread in rural areas of low-income countries [7, 8] where the lack of trained personnel and electricity prevents the use of traditional equipment.

With the development of portable and less expensive equipment, innovative projects showing benefits of ultrasound scans in rural areas are currently being implemented [8, 9]. However, further studies with larger samples are necessary to yield enough evidence.

We present here the results of the implementation of a new pregnancy control program (which is known as Healthy Pregnancy) aimed to reduce maternal and neonatal mortality in rural Guatemala. Therefore, it is important to first understand which the usual practice in rural Guatemala is.

### Current prenatal care program in rural Guatemala

Prenatal controls in isolated rural areas of Guatemala are achieved by community facilitators (CF), volunteers from a rural community who, having received basic training and a small stipend from the Ministry of Health, performs promotion and health care to a population of around 1,500 inhabitants. CFs are responsible for identifying pregnant women and for offering a basic prenatal control (weighing, measuring and checking blood pressure). The CFs are visited by health professionals (qualified nurses) approximately once a month, but nurses don’t have resources nor knowledge to perform additional medical test such as ultrasound scans or blood tests. This care program was part of the Extension Coverage Program (specific health program for rural areas, knows as PEC) of the Ministry of Public Health and Social Assistance (MSPAS according to its initials in Spanish) of Guatemala.

## Methods

We conducted an observational study that compares the impact achieved in maternal and neonatal mortality by the Healthy Pregnancy project versus the common practice in rural Guatemala (based on community facilitators). The intervention group for the Healthy Pregnancy initiative was decided by the Health Directorate of the Department of Alta Verapaz (Guatemala), who planned the travels of the nurses trying to reach the maximum amount of pregnant women with each visit.

**Fig. 1:**
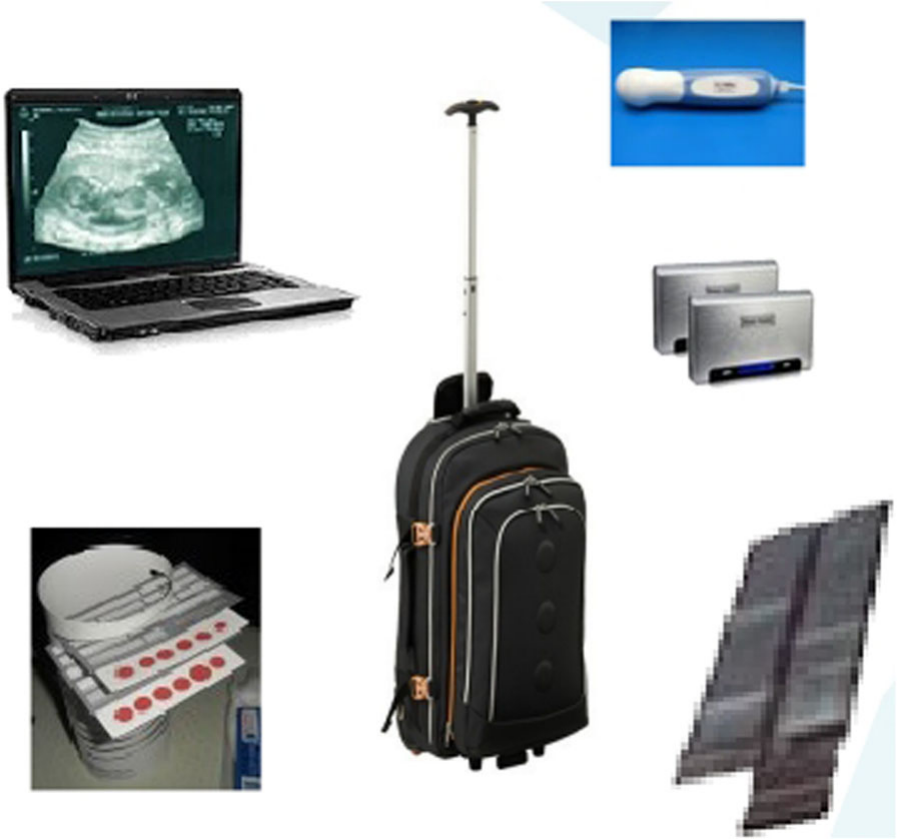
Prenatal care kit elements: laptop, USB ultrasound probe, dried blood screening tests, backpack, external batteries and folded solar panel

The Healthy Pregnancy project aimed the early detection of diseases and risk factors that could potentially result in maternal or neonatal deaths, by means of strengthening the prenatal care program for rural areas with a prenatal care kit (shown at Fig. 1), training and remote support. The prenatal care kit is composed by a portable ultrasound equipment (powered by external batteries and a small solar panel to work in isolate areas without electricity) and dried blood screening (DBS) tests (that do not require a cold chain or electricity for storage or transport). The prenatal care kit was complemented with the use of a health information system (www.medting.com) where the nurses uploaded the medical records of the prenatal attentions, including ultrasound images and blood and urine test results. This information system allowed gynecologists from Spain to check remotely the quality of the attentions and provide feedback to the nurses (in order to improve their training). Nurses are also provided with an intense one-week training on ultrasonography basics: checking fetal vitality, identifying the number of fetuses, evaluating gestational age, performing fetal biometry, measuring the amount of amniotic fluid, and identifying placental location and fetal presentation. The training includes theoretical lessons on prenatal care protocols and ultrasonography theory, and practical sessions with real pregnant women and the whole equipment to learn how to use an ultrasound probe. During practice lessons, each participant is closely supervised by the ultrasonography specialist who provides a personalized training and evaluates if required competences have been learned.

**Fig. 2:**
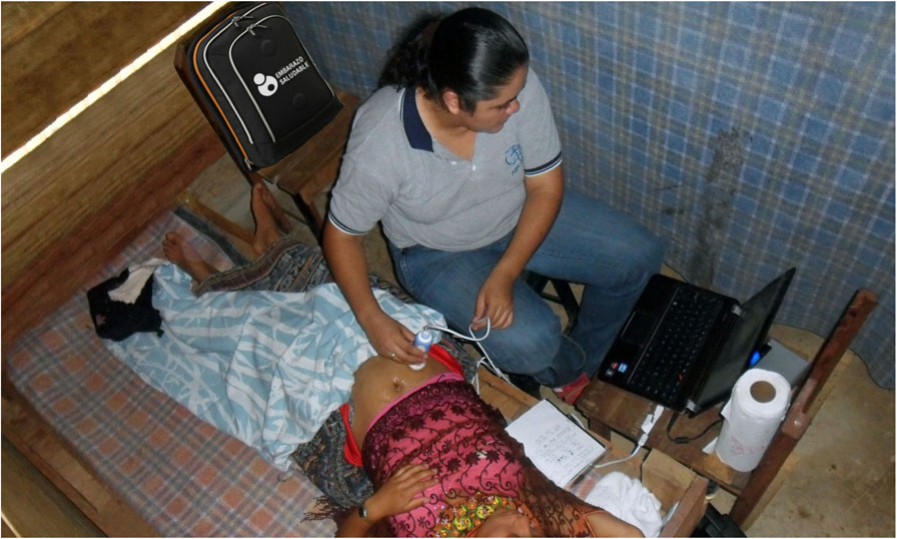
Nurse performing a prenatal control with the prenatal care kit

Between September 2012 and November 2013, the Healthy Pregnancy project strength the local prenatal care program providing three rural nurses with technology for ultrasonography and blood/urine tests (Fig. 2). The evaluation area was located in the districts of Senahu, Campur and Carcha (for both the intervention and the control groups), in the Department of Alta Verapaz. The department has about 1.2 million inhabitants; 78 % live in rural areas and indigenous population represents 89 % (the majority Q’ueqchi ethnicity). Among the indigenous population, 48 % live in extreme poverty and chronic child malnutrition reaches almost 60 %. In Alta Verapaz only 1 in every 3 births is attended by health workers. In 2012, 73 maternal death cases were reported out of 26,642 live births, resulting in a MMR of 274 (including urban areas).

The intervention group comprised pregnant women attended by the CFs, but with the visit of these 3 nurses trained and equipped to use the prenatal care kit. Thanks to the prenatal care kit, the intervention group received serological tests (HIV, HBV and syphilis), measurement of hemoglobin and glucose levels, urine dipstick tests and an ultrasonography study (valuation of fetal vitality, number of fetuses, gestational age, fetal biometry, pathology of amniotic fluid, placental location and fetal presentation). The visits of the nurses equipped with the prenatal care kit were scheduled by the Health Directorate of Alta Verapaz, seeking to maximize the number of ultrasound scans performed to women in the last trimester. The project protocol included a complete control (which included ultrasonography scans, and blood and urine tests) when pregnant women went to her first control (independently of the gestational age), and a second complete control at the third semester (from the week 32 and upwards). Between the two complete controls, the women should attend monthly basic controls provided by the CF, as defined in the official Guatemalan protocol.

The control group was composed of pregnant women from the same communities, but attended only by the CFs (the three nurses didn’t manage to reach all pregnant women). Even though the study is based on not randomized groups, and therefore, it is not possible to evaluate the statistical significance of the results, intervention and control groups are very homogeneous (income level, education, distance to a health facility, etc.) because they all come from the same communities and are attended by the same CF. The size of the both groups was determined by the number of possible visits during the intervention period.

Upon completing one year of the Healthy Pregnancy activities, we reviewed official records from the MSPAS on live births, stillbirths, and pregnant women deceased from any cause. Thereby, maternal and neonatal mortality rates were calculated in both groups. As secondary outcomes, the detection frequencies of each obstetric pathology and the referrals number were evaluated in the intervention group. Specific data on cases in the intervention group was mainly obtained from the information system previously mentioned.

## Results

From September 2012 to November 2013, the community facilitators attended a total of 1,509 pregnant women. Of these, 762 were visited by the nurses with the prenatal care kit (intervention group) and 747 were not (control group). See Table 1 for further detail on the structure of the sample, which was organized in three areas.

**Table 1:** Structure of control and intervention groups

	Pregnant women intervention group^a^	Pregnant women control group^b^	Total
Area A	219	338	557
Area B	262	157	419
Area C	281	252	533
Total	762	747	1509

^a^Data obtained from the project database

^b^Data obtained from the records of the health providers (PEC-MSPAS)

The first visit controls were performed to: 13 % of pregnant women under 14 weeks; 51 % at second trimester; and 36 % (122 women) above week 28. Only 104 women (out of 640, what means a 16 %) attended her second pregnancy control (above the 32 week) as scheduled. The second control was not scheduled for 122 women because they attended their first control after week 32. Summarizing, 226 controls were performed to pregnancies of 32 weeks or more.

### Maternal mortality

Whereas there were 5 maternal deaths in the control group, no maternal death cases were reported in the intervention group (MMR 0). Two of the five maternal deaths were due to postpartum hemorrhage (a case of retained placenta and a case of uterine atony), the third was a poisoning due to a suicide attempt (32 weeks of pregnancy), the fourth woman died due to emesis and aspiration (at term), and the last presented pneumonia during postpartum. Analysing each cause of death, the ultrasound scans and the test would have not provided risk indicators for the cases of aspiration, pneumonia, and suicide. However, it is reasonable to assume that the diagnosis and treatment of an anaemic patient could be useful for the haemorrhage cases, and this project helps to do that, which is especially important given the high prevalence of anaemia detected in the intervention group (42 %).

### Neonatal mortality

Respect neonatal mortality, official records of the MSPAS (supplied by the PEC) were used to analyse the impact in both groups (intervention and control) and are shown in Table 2. These records show 7 neonatal deaths in the intervention group, whereas in the control groups accounted for 19 cases. If we compare registered neonatal deaths in both groups, we found a 64 % reduction in neonatal mortality rate.

**Table 2:** Neonatal mortality for intervention group and control group

	Intervention group (*N* = 762 newborns)	Control group (*N* = 747 newborns)
Area A	1	2
Area B	4	11
Area C	2	6
Total	7 (9, 2 ‰)	19 (25, 4 ‰)

### Blood and urine tests

Blood tests were performed to 98.5 % of the pregnant women in the intervention group, detecting an anemia prevalence (Hb <11 g/dL) of 37 % (280 women), and identifying 42 cases (5.5 %) with hemoglobin levels under 9 g/dL. 633 results of urine dipsticks were reported, and urinary tract infection was diagnosed and treated in 29 cases (4.58 %). One patient was referred by positive proteinuria and suspected preeclampsia.

Although dried blood screening showed 2 HBV and 1 HIV positive cases, none of them were later confirmed by the central laboratory in Guatemala City.

### Patients referred to the hospital

In the intervention group, a non-urgent referral was recommended to 70 pregnant women (9.2 %) in order to receive attention at the reference hospital (Coban Regional Hospital). The main cause of reference was fetal malpresentation (66 % of cases), followed by multiple gestation, placenta previa and amniotic fluid problems (all detected by ultrasound). Thirty-two patients attended to the reference hospital (4 cases were transfers by emergency plan, and 1 case was referred for suspected twin pregnancy with posterior diagnosis of single pregnancy).

Not all the recommended referrals (70) were performed, twenty-two patients did follow nurse’s recommendations and followed its course in the community (cultural and economic reasons make it difficult for patients to move from rural areas to the city). Moreover, there is not childbirth information for 16 pregnant women to whom the reference was recommended, because the majority of them had an estimated due date after the data collection.

The reasons for the references and their final resolution are described in Table 3, where different referral reasons can belong to the same case (so the number of reasons is greater than the number of cases, 70). It is also important to note that this study only evaluates the implementation of technology to detect obstetric emergencies. The treatment provided once referral was recommended, depended on the protocols of the reference hospital.

**Table 3:** Referrals. Reason for referral, compliance and final pregnancy result

Reason for referral	N	Accepted references	Reference not performed	Lost^a^	Resolution
Suspected pathology in ultrasound (64 patients)					
First trimester abortion	1	1			Accepted: curettage.
Stillbirth (2^nd^ trimester)	2	1	1		Accepted: Induction and delivery.
					Not performed: delivery in the community.
Fetal macrosomia	1	1			Hospital birth. Healthy newborn.
Fetal malformation	2		1	1	Not performed: 37 weeks stillbirth, delivery in the community, fetal malformations confirmed.
Oligohydramnios^b^	1	1			Caesarean delivery (placenta previa). The newborn died (28 weeks).
Polyhydramnios	2	1	1		Both vaginal delivery and healthy newborns.
Placenta previa^b^	3	1	1	1	Accepted: Confirmed. Caesarean delivery. The newborn died (28 weeks)
					Not performed: vaginal delivery in the community.
Fetal malpresentation	46	18	18	10	Accepted: 18 healthy newborns.
					Not performed: 4 stillbirth
Twin pregnancy^c^	7	4	0	3	View discussion below.
Reason for referral	N	Pregnant attended	Pregnant did not attend.	Lost^a^	Resolution
Other suspected causes for referral (6 patients)					
Preeclampsia	2	2			Preeclampsia was confirmed. 1 vaginal delivery and 1 caesarean. Both healthy newborns.
Chronic hypertension	1			1	Lost: EDD later to data collection.
Positive screening test^c^	3	3			Negative confirmatory tests.
Age: 12 years old	1	1			Caesarean delivery due maternal age

^a^Expected delivery date (EDD) later to data collection, one case didn’t attend to the scheduled control

^b^Reference of a 28 weeks pregnant with 2 diagnosis: suspected oligohydramnios and placenta praevia

^c^Reference of a pregnant women with suspected twin pregnancy and positive serological screening test

Seven patients were referred for suspected twin pregnancy on ultrasounds:

- Three women were still pregnant at data closure.
- Gemellarity was not confirmed in one case.
- One patient had a 26 weeks delivery in hospital, but twins died.
- Of the 2 other cases with hospital birth, 3 healthy newborns were obtained and a stillbirth occurred.

#### Fetal malpresentation

Special relevance has the detailed review of the group referred due fetal malpresentation (Table 4). There were 46 referred cases (65.7 %), and data about the birth was available for 36 of them (the rest had an estimated due date later than the termination of data collection). Of these 36, one half went to the hospital for delivery and the other half gave birth at home. There were no neonatal deaths among the 18 who went to the hospital, whereas 4 neonatal deaths occurred among the 18 who did not accept the reference. This shows that the timely detection of fetal malpresentation and a correct referral have a significant benefit in neonatal mortality (the 64 % reduction in NMR previously shown would have been even higher if they would have accepted the reference).

**Table 4:** Neonatal deaths in the intervention group according to the reference response

Fetal malpresentation	Accepted references	Not performed references
At term	5 (5 Healthy NB/4 Cesarean/0 Death)	2 (0 Healthy NB/2 Death)
32–37 weeks	10 (10 Healthy NB/4 Cesarean/0 Death)	9 (7 Healthy NB/2 Death)
<32 weeks	3 (3 Healthy NB/0 Cesarean/0 Death)	7 (7 Healthy NB/0 Death)
Total	18 (0 Neonatal Death)	18 (4 Neonatal Death)

*NB* newborn

## Discussion

The care package here evaluated allowed to improve prenatal care in isolated rural communities (with energetic independence and without cold chain) through strategies, such as: screening for anemia, screening for maternal bacteriuria, screening for hypertensive disorders of pregnancy, iron supplementation to prevent maternal anemia and preparedness for births and emergencies. The portable ultrasound system (supplied with a foldable solar panel and external batteries) has also proved to be an important diagnostic tool for fetal malpresentation, twin pregnancy, amniotic fluid pathology and abortions. Through a basic ultrasonography study and blood and urine screening, pathology detection rate requiring non-urgent transfer (9.19 %) corresponds with literature [10, 11].

The results obtained in this study are consistent with previous findings by other researchers regarding the feasibility of training rural unskilled health workers on basic ultrasonography studies [11, 12] analysing fetal vitality, fetal biometry, placental localization and detection of twin pregnancies. Although the one-week training provided in this project allowed the nurses to start to perform ultrasound scans, we have noticed that this is the most demanding task and that bimonthly reinforcement trainings should be included in future phases of the project.

Moreover, thanks to the use of an information system, gynecologist have reviewed the attentions performed by the nurses, which has served to improve nurses training. However, an important limitation identified in the information system (with the version available on that year) was the lack of a report system to get statistics of the attentions. The reason was that the information system was designed for getting second opinions on medical cases (without caring about statistics), and therefore the cases upload had to be revised manually to get proper statistics. Future deployments will try to solve this problem by using information systems oriented to generate statistical analysis.

The introduction of these portable technologies as screening tools in rural areas, makes also necessary an improvement in personal and material resources, as well as in the coordination of work with local health agencies [10], in order to provide an optimal response to these references. However, availability and access to medical care are necessary but not sufficient factors to improve maternal and newborn health. In fact, they do not guarantee increased utilization of services. There is emerging evidence that increasing the access to and utilization of facility-based maternal care alone does not necessarily translate into better maternal outcomes [13].

Regarding the newborn, over 80 % of cases of fetal and neonatal deaths occur in pregnancies with complications [14]. Therefore, early detection and management of pregnancies with complications can improve maternal and perinatal outcome. In a recent WHO publication, it was shown that the prevalence of obstetric complications, severe maternal outcomes and perinatal mortality was higher in women with underlying indirect causes, being anemia the most common one [15]. This fact was verified in our intervention group, where a 37 % prevalence of anemia was detected thanks to the implemented tests. This is a relevant finding that will help to adapt prenatal care protocols of Guatemala to the specific problems of rural areas.

The project also revealed a hard reality: many women, despite being informed of the danger of childbirth in the community, rejected the referral. Even with a small number of women, analysing the perinatal outcome for fetal malpresentation in the reference group according to the acceptance or rejection of the reference, we found that there were a 22.2 % of neonatal deaths in the group of women who ignored the recommendation, versus 0 % in the group of women who attended to the hospital. This refusal is often related to economic difficulties but also reflects varying degrees of mistrust towards the traditional health system. The expansion of the project is likely to produce a greater awareness among the population on the use and benefits of ultrasonography, and to increase confidence and compliance of the people in rural areas toward the health system.

Impact data on maternal mortality (reduction to zero) and neonatal mortality (NMR was reduced to 36 %) are encouraging, although we are aware of the limitations of the study related to possible biasing and the small sample size. First, we need to note that the sample needed to conduct an experimental study of impact on maternal mortality rate (with a MMR close to 300 deaths per 100,000 live births) exceeds 30,000 women for both intervention and control group (calculated data to demonstrate a 35 % reduction with a 95 % security). Despite a sample of 1,000 women cannot offer “statistically significant” results, it has allowed us to get funding to extend the project to 10,000 women. Moreover, we have already mentioned the non-random assignment of pregnant women for intervention and control groups, with the potential biases that this may generate. Finally, although maternal mortality cases are well identified and registered in Guatemala, information on some neonatal deaths in rural areas could be being ignored or lost due to the isolation of certain communities, and this could generate an important level of under-reporting. However, as intervention and control groups have similar characteristics and have followed the same reporting procedure, it is reasonable to think that the under-reporting level would have been similar for both (control and intervention) groups.

Despite all these limitations, we believe the results of this study deserve to be presented to the scientific community, because we have not found clinical trials about the impact of ultrasonography on maternal or neonatal mortality in rural areas of developing countries, or about the combination of ultrasound scans and laboratory tests as part of antenatal care in those areas.

## Conclusion

In this study we have evaluated a portable ultrasound system and a system for blood and urine testing, combined with a specific training, which allows rural nurses to perform a good quality prenatal control, and to timely prevent most of obstetric emergencies.

The major reduction observed on maternal and neonatal mortality provides promising prospects for these low-cost diagnostic procedures. Although this study has substantial limitations for a global inference of its results, the scarcity of works assessing impacts in maternal and neonatal mortality is what makes this research relevant for those countries trying to reduce their maternal and neonatal mortality figures.

Currently we are expanding the number of rural brigades from 3 to 29 in order to reach more than 10,000 assessed pregnant women in Guatemala in the next two years. The results obtained following the new study will offer more accurate information about the impact of the proposed solution.

## Abbreviations

CF: Community facilitator
DBS: Dried blood screening
Hb: Hemoglobin
HBV: Hepatitis B Virus
HIV: Human Immunodeficiency Virus
MMR: Maternal mortality rate
MSPAS: Ministry of Public Health and Social Assistance
PEC: Extension Coverage Program
